# Use of Malaria Rapid Diagnostic Test to Identify *Plasmodium knowlesi* Infection

**DOI:** 10.3201/eid1411.080840

**Published:** 2008-11

**Authors:** Thomas F. McCutchan, Robert C. Piper, Michael T. Makler

**Affiliations:** National Institute of Allergy and Infectious Diseases, Bethesda, Maryland, USA (T.F. McCutchan); University of Iowa, Iowa City, Iowa, USA (R.C. Piper); Flow Incorporated, Portland, Oregon, USA (M.T. Makler)

**Keywords:** Borneo, lactate dehydrogenase, malaria diagnostic, monkey malaria, mimetopes, *Plasmodium cynomolgi*, *Plasmodium knowlesi*, dispatch

## Abstract

Reports of human infection with *Plasmodium knowlesi*, a monkey malaria, suggest that it and other nonhuman malaria species may be an emerging health problem. We report the use of a rapid test to supplement microscopic analysis in distinguishing the 5 malaria species that infect humans.

Recent reports of *Plasmodium knowlesi* infections in humans in Sarawak and Sabah in Borneo and in the Pahang Peninsula of Malaysia have focused attention on the potential of monkey malarias to be a human health issue (*1*,*2*). As much as 70% of malaria infections in regional hospitals in Borneo are the result of *P. knowlesi* infection; similar infections have been found in Thailand, the Philippines, and Singapore (*3*–*5*). To date, only patients in hospitals are being screened for the disease. To better understand the epidemiology of this apparent outbreak of *P. knowlesi* in humans, one needs a method to rapidly screen both monkeys and humans in areas of high disease prevalence, regardless of their present health status. Thus, a rapid test that could detect and distinguish among the primate malarias would not only benefit individual patients but would also provide an important epidemiologic tool to monitor the overall risk and prevalence of malaria.

We have known for nearly 8 decades that, under laboratory conditions, several monkey malarias are capable of infecting humans and that *P. knowlesi* can be transmitted to humans by mosquito bite (*6*,*7*). Work in Malaysia by a team from the National Institutes of Health nearly 50 years ago reported that transmission to humans was not occurring to any prevalent extent. Currently, we see major foci of the disease, which can be life-threatening. Although the current overall incidence of *P. knowlesi* infection in humans is low, an exacerbating problem is that it can be consistently misdiagnosed by microscopy as the more benign human malaria, *P. malariae* (*1*,*2*,*8*,*9*). The rapid replication rate of *P. knowlesi* and the resulting high level of parasitemia warrant immediate and aggressive treatment, whereas *P. malariae* does not. Although the use of PCR has been essential to defining the problem, a more rapid diagnosis would be an important tool for prompt medical treatment. Furthermore, incorporating the capability to detect *P. knowlesi* into existing rapid tests already capable of detecting the other 4 *Plasmodium* species that infect humans (*P. falciparum, P. vivax, P. ovale*, and *P. malariae*) would be beneficial.

*P. knowlesi* is transmitted by members of the *Anopheles leucosphyrus* group of mosquitoes that resides in the upper canopy of the forests in large areas of Southeast Asia; these *Anopheles* mosquitoes have infrequent contact with humans (*10*). With increasing encroachment into the forest areas to provide farmland, however, humans are likely to increase their exposure to this vector. The potential for *P. knowlesi* infection as well as other monkey malarias to expand into the human population is real. While the *P. knowlesi* parasite is carried by zoophilic mosquitoes, some monkey malarias such as *P. cynomolgi* and *P. inui* are transmitted by the same mosquito vectors that carry human malaria and therefore represent an even wider threat.

One important test developed for detecting human malarias is an antigen-capture test based on monoclonal antibodies (MAbs) to plasmodium lactate dehydrogenase (pLDH). The 4 human malarial LDH isoforms have been cloned, and >20 MAb have been raised that differentially recognize epitopes among the isoforms (*11*,*12*). The specificity of a subset of these antibodies is shown in Figure 1. Of the 4 human *Plasmodium* spp., antibodies such as 17E4 and 7G9 specifically bind only to *P. falciparum* LDH, whereas antibodies such as 11D9 and 13H11 bind only to *P. vivax* LDH.

**Figure 1:**
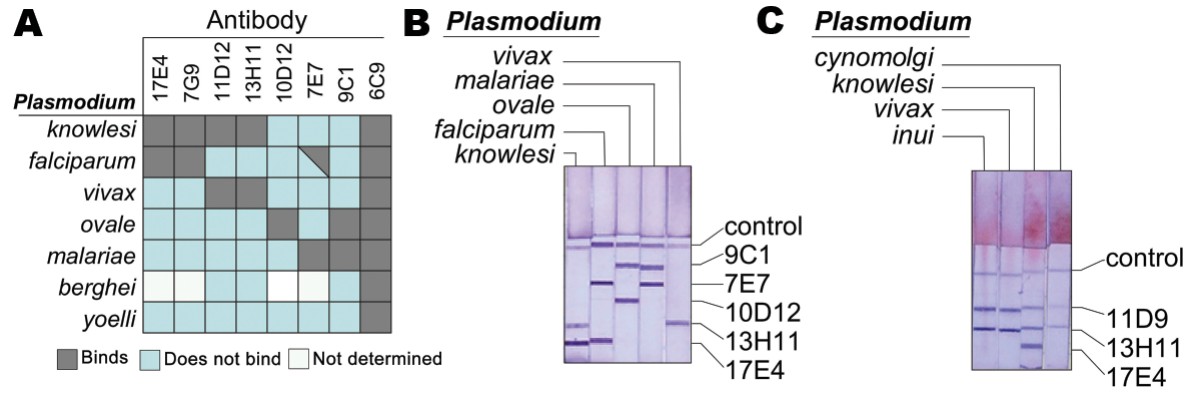
Binding specificity of different anti–*Plasmodium* lactate dehydrogenase (pLDH) antibodies. A) Shown are the reactivities of the indicated monoclonal antibodies (MAbs) to the LDH from 7 *Plasmodium* spp. Reactivity was determined by using an immunocapture assay as previously described (*9*). B) Example of an immunodipstick assay that detects *P. knowlesi*. An immunochromatographic strip assay containing the indicated antibodies was allowed to wick lysed blood infected with *P. vivax, P. falciparum*, *P. knowlesi, P. ovale*, or *P. malariae.* Blood was wicked in the presence of colloidal gold conjugated to antibody 6C9, which binds all pLDH isoforms. *P. vivax* LDH is immobilized only by 11D9 and 13H11, and *P. falciparum* LDH was only immobilized by 17E4. *P. knowlesi* LDH was immobilized by 11D9 and 13H11 antibodies and also by 17E4. C) An immunochromatographic strip assay containing the indicated antibodies was allowed to wick lysed blood infected with *P. vivax, P. cynomolgi, P. inui,* and *P.* *knowlesi*. Blood was wicked in the presence of colloidal gold conjugated to antibody 6C9, which binds all pLDH isoforms. Both *P. cynomolgi* and *P. inui* show the same epitope profile as *P. vivax*.

Using this panel of antibodies, we show that we can distinguish *P. knowlesi* from *P. malariae*. *P. knowlesi* binds to both the “falciparum-specific” (17E4/7G9) and the “vivax-specific” (11D9/13H11) antibodies (Figure 1, panels **A** and **B**). Furthermore, *P. knowlesi* does not react with 10D12 (an antibody specific for *P. ovale*), 7E7 (an antibody that reacts strongly with *P. malaria*e and weakly with *P. falciparum*), or 9C1 (an antibody that reacts exclusively with *P. ovale* and *P. malariae*). Detecting *P. knowlesi* in monkeys, which often are co-infected with several other malaria parasites, is also important and can be achieved with the same panel of antibodies. We have tested the reactivity of the *P. falciparum*–specific antibody (17E4/7G9) with the other monkey malarias known to be indigenous to Malaysia (*P. cynomolgi*, *P. inui,* and *P. fieldi*) and found that none react (Figure 1, panel **C**). This then serves as a basis for distinguishing *P. knowlesi* from the other prevalent forms of monkey malaria.

The unexpected pattern of antibody recognition on which we based our tests led us to examine the molecular basis of recognition (Figure 2). As expected, *P. knowlesi* LDH is highly similar to the known pLDH isoforms. We found that only a few residue differences could account for the epitope differences detected by the 17E4/7G9 and 11D9/13H11 antibodies. We first generated a 3-dimensional model of *P. knowlesi* LDH and then mapped surface-exposed residues that were uniquely shared by *P. falciparum* or *P. vivax* isoforms. The protein structure was calculated by using the structures of *P. falciparum* and *P. vivax* LDH (PDB: 2A94 and 2AA3) and the WURST threading server (*13*). Here, only a few patches of residues were found to describe the *P. vivax*–specific epitope, and only 1 residue (K115) was found to describe the *P. falciparum*–specific epitope (Figure 2, panel **B**). Thus, the existing MAbs perform well at distinguishing pLDH isoforms despite only a small number of different surface-exposed residues.

**Figure 2:**
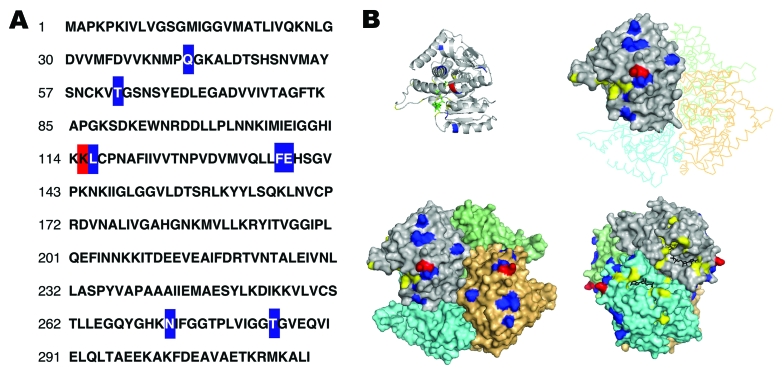
Modeling of the analysis of *Plasmodium knowlesi* lactate dehydrogenase (LDH). A) Sequence of LDH from *P. knowlesi* deduced from genomic DNA fragments sequenced by the Sanger malaria genome project (www.sanger.ac.uk/Projects/P_knowlesi). LDH isoforms from *P. vivax, P. malariae, P. ovale, P. berghei, P. yoelli*, and *P. falciparum* were compared with that of *P.* *knowlesi*. Residues unique to *P.* *knowlesi* and *P.* *vivax* are shown in blue; residues unique to *P.* *knowlesi* and *P.* *falciparum* are shown in red. B) Model of *P. knowlesi* LDH and specific epitopes. A model for *P. knowlesi* LDH was calculated by using WURST protein threading server (www.zbh.uni-hamburg.de/wurst/index.php) and the *P. falciparum* and *P. vivax* crystal structures (PDB: 2A94 and 3 2AA3). Shown is the monomer, as well as the assembled tetramer, aligned to the backbone of the *P. vivax* tetramer using pymol. The nicotinamide adenine dinucleotide cofactor analog 3-acetyl pyridine adenine dinucleotide is shown in black. Residues important for substrate binding and catalysis are shown in yellow. *P. knowlesi* residues shared only with *P. vivax* are shown in blue and indicate where the 11D9/13H11 epitopes could be. *P. knowlesi* residues shared only with *P. falciparum* are shown in red and indicate a critical determinant of the 17E4/7G9 epitopes.

These data show that pLDH antibodies that detect *P. falciparum* and *P. vivax* can also be used to detect and distinguish *P. knowlesi.* The 1 major caveat is that a *P. knowlesi* infection cannot be distinguished from a mixed infection with both *P. vivax and P. falciparum* in the blood. Mixed infections of this description, however, are infrequent, as these species do not proliferate concurrently when both are present in the blood (*14*,*15*). Furthermore, any confusion would be resolved by microscopic examination of blood that, while inadequate to distinguish *P. knowlesi* and *P. malariae*, would serve to distinguish *P. knowlesi* from mixed infections.

Obviously, an antibody specific for *P. knowlesi* would be optimal if the threat of *P. knowlesi* increases. Although development of a specific antibody would be a considerable investment, our epitope analysis discussed here indicates that only small sequence differences in pLDH isoforms are required to generate antibody panels capable of uniquely distinguishing animal pLDH isoforms.
